# QSAR with experimental and predictive distributions: an information theoretic approach for assessing model quality

**DOI:** 10.1007/s10822-013-9639-5

**Published:** 2013-03-16

**Authors:** David J. Wood, Lars Carlsson, Martin Eklund, Ulf Norinder, Jonna Stålring

**Affiliations:** 1 Novartis, Horsham, UK; 2 AstraZeneca, Mölndal, Sweden; 3 Lundbeck, Copenhagen, Denmark

**Keywords:** Quantitative structure–activity relationships, QSAR, Kullback–Leibler divergence, Predictive distributions, Applicability domain, Prediction errors, Prediction confidence

## Abstract

We propose that quantitative structure–activity relationship (QSAR) predictions should be explicitly represented as predictive (probability) distributions. If both predictions and experimental measurements are treated as probability distributions, the quality of a set of predictive distributions output by a model can be assessed with Kullback–Leibler (KL) divergence: a widely used information theoretic measure of the distance between two probability distributions. We have assessed a range of different machine learning algorithms and error estimation methods for producing predictive distributions with an analysis against three of AstraZeneca’s global DMPK datasets. Using the KL-divergence framework, we have identified a few combinations of algorithms that produce accurate and valid compound-specific predictive distributions. These methods use reliability indices to assign predictive distributions to the predictions output by QSAR models so that reliable predictions have tight distributions and vice versa. Finally we show how valid predictive distributions can be used to estimate the probability that a test compound has properties that hit single- or multi- objective target profiles.

**Electronic supplementary material:**

The online version of this article (doi:10.1007/s10822-013-9639-5) contains supplementary material, which is available to authorized users.

## Introduction

Models of quantitative structure-activity relationships (QSARs) are widely used throughout the pharmaceutical industry to predict the pharmacological properties of virtual compounds and to guide the selection of compounds for synthesis [1]. However, it is impossible for a drug discovery scientist to know the extent to which a QSAR prediction should influence a decision in a project unless the expected error on the prediction is explicitly and accurately defined [2, 3]. A QSAR model can only be expected to provide reliable predictions for test compounds that fall within the model’s Applicability Domain (AD), although the AD is often a difficult property to define. The OECD guidelines for QSAR modeling recognize that typically there is no absolute boundary between reliable and unreliable predictions, and that setting a model’s AD requires a tradeoff between the constraints of the model and the accuracy of its predictions [4]. The AD can therefore be thought of as a gradual property of the model space, and estimations of expected error that are provided with QSAR predictions should reflect the degree to which the test compounds fall into the AD.

A number of different reliability indices have been proposed for the definition of ADs [5]. Distance-to-model metrics are the most extensively studied and represent some measure of the distance between a test compound and the compounds used in the model’s training set [6–9]. Test compounds with high similarity to the training set compounds are assumed to produce more accurate predictions than dissimilar test compounds. Alternative approaches involve defining regions of the descriptor space with different levels of reliability [10, 11], or assessing the sensitivity of a model’s predictions to small changes in the input data, either by perturbing the input descriptors or with a process known as bootstrap aggregating [12]. These reliability indices generally serve as proxies to prediction errors and can either be used to indicate when predictions should not be trusted [12], or preferably, can be mapped onto a quantitative estimation of error that allows easy interpretation by the model users [7, 9, 12, 14].

Given that QSAR predictions should consist of both a point prediction and a quantitative estimation of error, in our opinion QSAR predictions should be explicitly defined as probability distributions. A ‘Predictive Distribution’ is a representation of a QSAR prediction that describes the probability that a test compound has a particular property value across a range of possible values. There are a number of advantages to representing QSAR predictions as predictive distributions: errors are intrinsic to predictive distributions and must be explicitly defined; it is straight forward to derive confidence intervals from predictive distributions, which are probably the most intuitive representation of errors for drug discovery scientists; and predictive distributions can be used to estimate the probability that an untested compound has properties that match a target property profile.

Sahlin et al. [15] recently summarized approaches towards the definition of Predictive Distributions used in the field of QSAR. Most approaches assume that the distribution of prediction errors has a functional form, for example, a Gaussian distribution. In work describing QSAR models for environmental toxicity, Tetko et al. [9] assumed Gaussian prediction errors and assigned different error variances to prediction queries according to distance-to-model criteria. Probabilistic modeling approaches, such as conditional density estimators and Bayesian models, output explicit probability distributions, and Gaussian Process Regression is one example of a Bayesian approach that has been applied to QSAR [16–18]. Probabilistic approaches have otherwise received little attention within the field of QSAR, perhaps because they are computationally intensive and unsuitable for datasets of the size frequently considered by pharmaceutical companies.

Most pharmaceutical assays have a non-negligible measurement error, and the experimental measurements used to generate and test QSAR models should therefore also be treated as probability distributions. If both the experimental data and the QSAR predictions are represented in this way, the quality of a test set of predictive distributions obtained from a QSAR model can be assessed with Kullback–Leibler divergence: an information theoretic measure of the distance between two probability distributions [19, 20]. In this paper we outline a framework for assessing predictive distributions output by QSAR models. Using this framework, we have assessed a range of different machine learning algorithms and error estimation methods against three of AstraZeneca’s global datasets: Caco2 Permeability, Human Plasma Protein Binding and LogD_7.4_. We report the results of these studies, and we demonstrate how the predictive distributions output by the models can be used to calculate the probability that a compound has properties that hit both single- and multi-objective target profiles.

## Methodology

Our framework for assessing QSAR predictions as probability distributions is based upon KL divergence. As an initial step, we have assumed that all prediction and experimental measurement errors are Gaussian distributed, although it should be emphasized that this is not a fundamental requirement for the approach. Under the Gaussian assumption, all data points are represented by two parameters *μ* and *σ*, where *μ* represents the traditional data point values used in QSAR analyses and *σ* represents the standard deviation of the (predictive) error distribution. A QSAR model must therefore comprise a model that provides the prediction values (*μ*) and a method that assigns quantitative error estimates (*σ*) to the predictions. In this paper we will refer to the models that provide prediction values as *models*, and the methods for estimating prediction errors as *error estimation methods*. The combinations of *model* and *error estimation method* that are required to produce the predictive distributions are referred to as *Predictive Distribution (PD) methods*.

### Kullback–Leibler divergence

The Kullback–Liebler (KL) divergence is a fundamental property in information theory that quantifies the distance of a modeled or hypothesized probability distribution, *Q*, from a true, underlying probability distribution, *P*. It is the inverse of Boltzman entropy and is a natural criterion for model selection within a maximum likelihood framework [19]. It therefore forms the basis of a wide range of information criteria for the selection of parsimonious models, including the Akaike or Bayesian information criteria (AIC or BIC) [21, 22]. While there are numerous alternative methods for assessing the distance between probability distributions [23], we have chosen to use KL divergence because of its fundamental role in maximum likelihood theory and because it is probably the most widely used metric for comparing probability distributions. Within the field of cheminformatics, Nisius et al. [24] used KL divergence to reduce the dimensionality of molecular fingerprints for similarity searching. For probability distributions *P* and *Q* of a continuous random variable *x,* the KL divergence is calculated with Eq. 1, where *p(x)* and *q(x)* are the densities of *P* and *Q* at point *x*.

1 $$ D_{KL} \left( {P,Q} \right) = \mathop \int \limits_{ - \infty }^{\infty } p\left( x \right)\ln \frac{p\left( x \right)}{q\left( x \right)}dx $$

For QSAR validation studies, given a test set of compounds with associated measurements that are represented as probability distributions, KL divergence can be used to quantify the information content of a set of predictive distributions [20]. Each experimental measurement, with an associated error, represents the true probability distributions, *P*, and a predictive distribution represents the modeled probability distribution, *Q*. Given two Gaussian shaped probability distributions—a true distribution, *P* = *N*(*μ*_*q*_, *σ*_*q*_), and a model distribution, *Q* = *N*(*μ*_*q*_, *σ*_*q*_)—KL divergence is calculated with Eq. 2.

2 $$ D_{KL} \left( {P,Q} \right) = 0.5\left\{ {\frac{{\left( {\mu_{p} - \mu_{q} } \right)}}{{\sigma_{p}^{2} }} + \ln \frac{{\sigma_{p}^{2} }}{{\sigma_{q}^{2} }} + \frac{{\sigma_{q}^{2} - \sigma_{p}^{2} }}{{\sigma_{q}^{2} }}} \right\} $$

The divergence is minimized when the mean of the model distribution equals the mean of the true distribution (*μ*_*p*_ = *μ*_*q*_) and when the variance of the model distribution equals the variance of the true distribution (*σ*_*p*_ = *σ*_*q*_). It should be noted that because our experimental measurements represent the ‘true’ probability distribution, predictive distributions from a QSAR model are penalized when they are more accurate and precise than the corresponding experimental measurements. This is because, within the KL framework, a predictive distribution represents the likely result of an experimental measurement, rather than the intrinsic property value for the molecule. In this sense, a model cannot predict an experimental result more precisely than the error on the measurement. As a practical step, we have set a lower bound to the prediction errors: if an *error estimation method* suggests an error that is lower than experimental error, it is re-assigned a prediction error that is equal to the experimental error.

Given a test set of *N* predictive distributions, $S_{Q} = \left\{ {\mu_{{q_{i} }} ,\sigma_{{q_{i} }} } \right\}_{i = 1}^{N}$, and an associated set of experimental measurement distributions, $S_{P} = \left\{ {\mu_{{p_{i} }} ,\sigma_{{p_{i} }} } \right\}_{i = 1}^{N}$, the mean of the divergences provides a measure of the total entropy (or inverse information) of the set of predictive distributions (Eq. 3).

3 $$ KL_{AVE} \left( {S_{P} ,S_{Q} } \right) = \frac{1}{N}\mathop \sum \limits_{i = 1}^{N} D_{KL} \left( {S_{{P_{i} }} ,S_{{Q_{i} }} } \right) $$

When comparing sets of predictive distributions output by different models applied to a common test set, the model with the lowest *KL*_*AVE*_ can be considered to have maximized information and should be used to make any future predictions on unseen examples. A model that results in a low *KL*_*AVE*_ has delivered predictive distributions that are accurate and that properly represent the uncertainty associated with the predictions. Conversely if the predictive distributions output by the model are inaccurate, inappropriately precise or unnecessarily imprecise, this will be reflected by a higher *KL*_*AVE*_ score. KL divergence has some advantages over metrics derived from residual errors such as Root Mean Squared Error (RMSE) or Q^2^. For example, the two, often competing modeling objectives of (1) *accuracy of predictions* and (2) *accuracy of error estimates* become a single objective: the information content of the predictive distributions output by a model. This avoids the need for subjective decisions on which of these two objectives is of greatest importance when comparing candidate models with differing attributes.

**Fig. 1:**
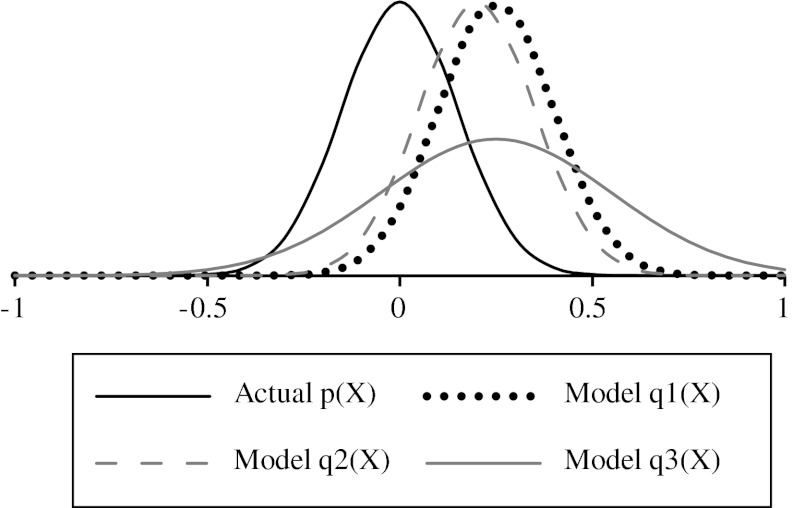
Example calculation of KL divergence

Figure 1 provides a demonstration of the calculation for a single test compound. Three probability distributions representing QSAR predictions from different models (*Q*_1_, *Q*_2_, *Q*_3_) are compared to the experimental probability distribution (*P*), and the *μ* and *σ* values for each of the distributions are provided in Table 1. The KL divergences for the three models are 1.4, 0.9 and 0.7, respectively. Distribution Q_2_ (shown as a dashed grey line) is the most accurate with a residual error of 2.0 compared to 2.5 for the other two models; however, the standard deviation (*σ*_*q*_) is too low to comfortably cover the full range of possible values represented by the true distribution, *P*. In other words, the error estimate assigned to predictive distribution Q_2_ is too low. As a consequence, predictive distribution Q_3_, which has a larger standard deviation and covers the full range of possible values, has the lowest KL divergence. From this point on, to aid readability, we will switch from the standard statistical notation of *N*(*μ*_*p*_, *σ*_*p*_) and *N*(*μ*_*q*_, *σ*_*q*_) to *N*(*μ*_*obs*_, *σ*_*obs*_) for measurement distributions and *N*(*μ*_*pred*_, *σ*_*pred*_) for predictive distributions.

**Table 1:** Parameter values for the example calculation of KL divergence

	*Μ*	*σ*	KL
*P*	0.0	1.0	–
*Q*_1_	2.5	1.5	1.4
*Q*_2_	2.0	1.5	0.9
*Q*_3_	2.5	3.0	0.7

### Measurement probability distributions

The measurement data-points used to train and validate the models are represented by measurement probability distributions that are defined by two parameters: *μ*_*obs*_ and *σ*_*obs*_. The mean value (*μ*_*obs*_) is the mean measurement for the compound and is the value traditionally used in QSAR analyses. The standard deviation (*σ*_*obs*_) represents an estimate of the error on the mean and is calculated with Eq. 4, where *σ*_*exp*_ is the ‘single-shot’ measurement error for the assay and *N* is the number of measurements for the compound.

4 $$ \sigma_{obs} = \frac{{{{\upsigma}}_{ \exp } }}{\sqrt N } $$

For each of the assays, we estimated *σ*_*exp*_ by analysis of the measurement variance for quality control (QC) compounds, which are run through the assays every day to check the consistency of the results. We used the QC compounds to assess experimental error, rather than using all compounds with more than 1 measurement, because experiments are most likely to be repeated when the measurement is suspected to be incorrect because of a problem with the initial experiment. Given a set of *M* quality control compounds, $\left\{ {QC_{i} } \right\}_{i = 1}^{M}$, each of which has $N_{{QC_{i} }}$ associated experimental measurements, $QC_{i} = \left\{ {QC_{ij} } \right\}_{j = 1}^{{N_{{QC_{i} }} }}$, we calculated the *σ*_*exp*_ value for the assay with Eq. 5.

5 $$ {{\upsigma}}_{ \exp } = \sqrt {\frac{1}{{\mathop \sum \nolimits_{i = 1}^{M} N_{{QC_{i} }} }}\mathop \sum \limits_{i = 1}^{M} \mathop \sum \limits_{j = 1}^{{N_{{QC_{i} }} }} \left( {QC_{ij} - \overline{{QC_{i} }} } \right)^{2} } $$

We have assumed that the measurement errors for all non-QC compounds are the same as the errors observed on the QC compounds. There are between 1 and 5 QC compounds for each of the endpoint datasets used in this work.

### Predictive distributions

The *PD methods* produce Gaussian-shaped predictive distributions, *N*(*μ*_*pred*_, *σ*_*pred*_). The mean values (*μ*_*pred*_) are the predictions obtained from models, which were generated using AstraZeneca’s AutoQSAR system [25]. We used 4 different machine learning algorithms that are available in R (v2.14.0): [26] Partial Least Squared (PLS); k-Nearest Neighbours (KNN); Random Forests (RF); and Support Vector Machines (SVM).

PLS creates linear models from principal components of the input data. The models were generated with the R *pls* library [27] using the approach previously described in Wood et al. [25]. RFs are ensembles of regression trees each built with a different bootstrap sample of the training data. RF models were generated with the R *random Forest* library [28]. Forests comprised of 250 trees, and the parameter *nodesize*, which specifies the point at which tree nodes are not further split into child nodes, was set to the default value of 1. The parameter *mTry* specifies the size of the random subset of descriptors consider for each node split and was optimized against the training set out-of-bag error using the *tuneRF* method. The KNN algorithm predicts properties of test compounds from the *k* nearest neighbors in a training set of examples. Distance weighted KNN models were generated using the R library *kknn*. The triangular kernel was used in all cases, and the parameter *k*, which represents the number of nearest neighbors used to form the predictions, was optimized with a sevenfold cross validation on the training set. SVM models were generated with the R *e1071* implementation of the LIBSVM algorithm. [29, 30] The SVMs were constructed with a Gaussian Radial Basis Function kernel. The optimal value for the parameter γ was identified from the set {2^−8^, 2^−7^, 2^−6^} and the cost parameter was set to 2 [3]. The optimal parameter values were identified with a grid search within this limited parameter space using sevenfold cross validation.

All compounds were represented by the ‘AZ’ descriptor set, which comprises 193 descriptors that include physicochemical properties, topological indices and structural counts. The descriptors were calculated with the following software packages: *ACD/PhysChem Suite* [31], *HYBOT* [32], and AstraZeneca in-house descriptor package*, SELMA* [33]. We have provided the full list of descriptors in the supplementary information.

#### Assigning prediction errors, *σ*_*pred*_

The value *σ*_*pred*_ represents an estimation of the expected error of a prediction. Under the Gaussian assumption we can expect the true property value to lie within ±1 *σ*_*pred*_ of the prediction value for 68 % of predictions, and within ±2 *σ*_*pred*_ for 96 % of predictions. We have used two general approaches for setting *σ*_*pred*_. Uniform error estimation methods assume that the expected prediction error is the same for all test compounds regardless of any judgments on their reliability. Variable error estimation methods assign compound-specific prediction errors with the aid of a reliability index. The uniform methods serve as null hypotheses in these experiments: to be of practical use, any method that assigns compound-specific prediction errors must represent an improvement in information relative to the uniform methods.

We used two different uniform error estimation methods. The temporal Test Set (TS) method assesses the accuracy of the models’ predictions with the large ‘parameterization’ test sets that are described below. The Cross Validation (CV) method uses double loop cross-validation on the models’ training sets to determine the expected error of future predictions. Double loop cross validation techniques are used to ensure that the estimations of the models’ generalization errors are not biased by the parameterization of the models [34, 35]. For both uniform error estimation methods, the *σ*_*pred*_ value for all future predictions is set to be equal to the Root Mean Squared Error (RMSE) of the predictions produced by the validation procedure.

We investigated a range of different reliability indicators for setting compound-specific (variable) prediction errors, which are described below.

#### Distance-to-model (D2M)

Distance-to-model approaches to the estimation of prediction reliability have been widely reported in the literature. [6–14] Given a distance function, the average distance of a test compound to the *k* nearest training set neighbors indicates the relative reliability of the prediction. We calculated distances with the Euclidean and Mahalanobis distance functions on the input descriptor space, with all descriptors scaled to zero-mean, unit variance. The Euclidean distance between two vectors A and B is calculated with Eq. 6.

6 $$ D_{EUC} \left( {A,B} \right) = \sqrt {(A - B)(A - B)^{T} } $$

The Mahalanobis distance function is given in Eq. 7, where S^−1^ is the estimated inverse covariance matrix for the training data. The inverse covariance matrix could not be solved exactly for our training data matrices and was approximated using singular value decomposition with the R library *MASS.* [36].

7 $$ D_{MAL} \left( {A,B} \right) = \sqrt {\left( {A - B} \right)S^{ - 1} \left( {A - B} \right)^{T} } $$

**Fig. 2:**
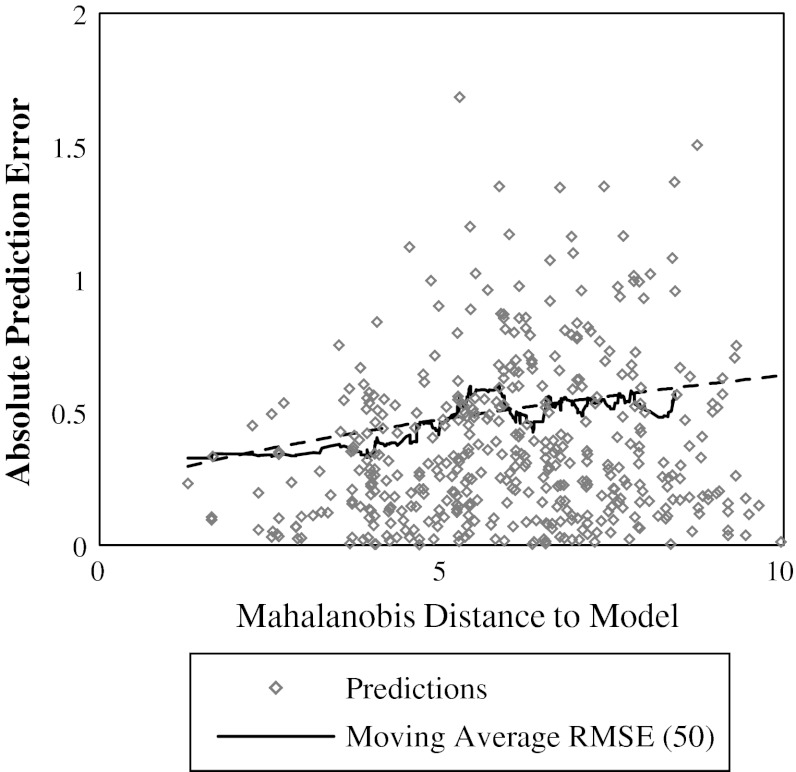
Converting distance-to-model to estimations of prediction error

The resulting distance-to-model values are converted to estimates of the expected prediction error (*σ*_*pred*_) by finding a linear regression between distance and the residual squared errors for predictions from the parameterization test sets. The *y* intercept for the regression was fixed to the square of the experimental error for the assay so that *σ*_*pred*_ is equal to the experimental error for distances of zero. Figure 2 shows an example regression using the Caco2 parameterization test set with the mean Mahalanobis distance to the nearest 3 neighbors (MD_3_) used as the reliability index. The black line shows the moving average RMSE with a block size of 50. The regression line $\left( {\sigma_{pred} = \sqrt {0.037D_{MAL\_3} + 0.04} } \right)$ fits the moving average well. The regression parameters and the parameter *k* were optimized with the parameterization test sets (described below), and the optimal values were used for all subsequent ‘future’ predictions.

#### Local error

The local error (LE) approach estimates the value of *σ*_*pred*_ to be equal to the RMSE of the double loop cross validated predictions for the nearest *k* neighbors in the model’s training set. The underlying assumption is that, if a test compound is similar to training set compounds that were poorly predicted, the model is likely to perform poorly on the test compound. Again, we used the Euclidean and Mahalanobis distances within the scaled input descriptor space to identify the nearest training set neighbors. We tested an additional corrected local error (LEC) approach where a regression analogous to the one described above for D2M is used to transform the initial local error estimate into a final, corrected estimation of the expected error.

#### Bagged variance

The bagged variance (BV) method has recently been shown to be a very effective reliability indicator for QSAR predictions [12, 15]. The method requires that ensembles of models are generated with a *bagging* procedure, where individual models are built with different, randomly generated bootstrap samples of the training data set [37]. The standard deviation of the individual predictions for a test compound across the ensemble is an indicator of the reliability of the prediction for the compound. A large standard deviation in the individual predictions indicates that the model is not stable for the compound and corresponds to greater expected prediction error. The BV indicator is easily calculated from Random Forest models as they are already bagged ensembles of regression trees. Furthermore, because each individual tree in the forest is a low bias, high variance representation of the training data, the standard deviation of the predicted values across the ensemble can serve as a direct estimation of the expected prediction error. Generating bagged ensembles for the other machine learning algorithms considerably extended the models’ training times and proved unfeasible for the SVM algorithm, as it takes several days to train a single SVM model with these large global datasets. However, we generated bagged variations of the PLS and KNN algorithms by applying the algorithms to 100 bootstrap samples of the training data. We set up two different error estimation methods based on Bagged Variance. The uncorrected Bagged Variance (BV) method uses the standard deviation of the predictions across the ensemble as a direct estimation of the expected prediction error, *σ*_*pred*_, whereas the Corrected Bagged Variance (BVC) approach finds a linear regression of bagged variance to the expected prediction error using the same method as described above for D2M.

#### Error model

In this approach, prediction errors are estimated with a second model generated with machine learning. We used the R PLS algorithm to model the absolute errors of the predictions produced by the double loop cross validation. The resulting *PD models* consist of a model that predicts the property values (using KNN, PLS, RF or SVM) and a model that estimates the errors on those predictions (using PLS).

### Datasets and experimental methodology

We assessed the *PD methods* described above with three global AstraZeneca datasets: LogD, Human Plasma Protein Binding, and Caco2 A to B permeability. LogD data were generated with a shake flask methodology described by Wenlock et al. [38] Caco2 A to B Permeability (Caco2) was measured across 2-week old Caco2 cell monolayers in a pH-gradient system, as described in essence by Neuhoff et al. [39] The Caco2 data were modeled in units of log cm/s. Human Plasma Protein Binding data (hPPB) were generated with an assay previously described by Leach et al. [40] and were modeled in units of log bound/free. Any experimental measurements in our datasets that were annotated with a comment that suggested an issue with the experiment were removed. We treated qualified data points, where the experimental measurement is indicated to be greater or less than a specified value, as quantitative measurements by ignoring the qualifying symbols. We have found treating qualified data points in this way results in improved prediction accuracies relative to the alternative approach of removing all qualified data from the models’ training sets.

**Fig. 3:**
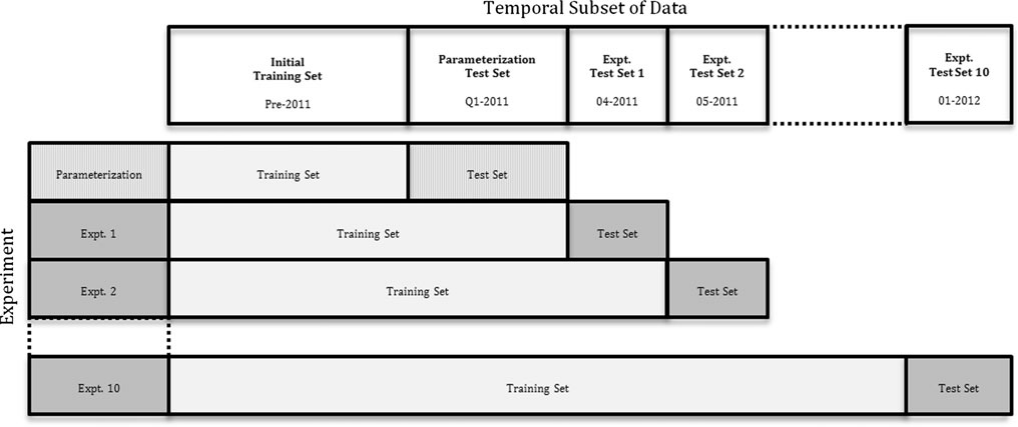
Schematic of the experimental methodology. Temporal subsets of the data are represented by columns and the model building/prediction experiments are represented by rows. The parameterization test set (shown in *light textured grey*) is used to set the parameters for the error estimations methods, whereas the experimental test sets (shown in *solid grey*) are used to assess the performance of the various PD methods. There are 10 experimental test sets in total which are predicted by models built on growing training sets

Our experimental methodology is shown schematically in Fig. 3. We began by dividing each of the datasets into a series of temporal subsets as shown in Table 2. For each endpoint, the first subset comprised all data collected before 2011 and provided an initial training set for the models. The second subset for each endpoint included all data collected during the 1st quarter of 2011 and served as a ‘parameterization’ test set that was used to optimize any parameters required by the error estimation methods. The 10 months from April 2011 to January 2012 represent 10 experimental test sets that provided ‘future’ tests for the *PD* *methods*. These experimental test sets are intended to represent the kind of compounds that would be applied to models like these in real life use. They provide unbiased tests for the models as they were not used in the model optimization and parameterization processes.

**Table 2:** Numbers of compounds in the global datasets

Dataset	Period	LogD	hPPB	Caco2
Initial training set	Pre-2011	34,837	34,450	13,037
Parameterization test set	Q1-2011	2,457	1,586	451
Experimental test sets	04-2011	879	649	68
	05-2011	765	491	171
	06-2011	923	293	178
	07-2011	848	451	80
	08-2011	932	517	64
	09-2011	866	503	109
	10-2011	780	415	249
	11-2011	392	426	278
	12-2011	739	639	189
	01-2012	984	408	84
Combined expt. test sets	04-2011 to 01-2012	8,108	4,792	1,470

To minimize the computational resource requirements for these experiments, we reduced in size any training dataset that contained more than 35,000 compounds by selecting a subset that consisted of the most recent 20,000 compounds and a random selection of 15,000 of the remaining compounds. We have found that this process has only a marginal effect on the predictive performance of the models. The number of compounds included in each of the datasets is given in Table 2, with the initial training set numbers representing the reduced dataset sizes.

For each endpoint, we generated an initial QSAR model using each of the 4 machine learning approaches described above. These initial models were applied to the initial parameterization sets, and the resulting sets of predictions were used to optimize the parameters of the various error estimation methods. We then combined the initial parameterization test sets with the initial training sets, and the models were rebuilt and used to generate predictions for the first experimental test set (April 2011), with prediction errors estimated with the previously parameterized error estimation methods. This process was repeated until predictions were obtained for all experimental temporal test sets with updating QSAR models. Finally, we combined the predictions of the individual experimental test sets and used them to assess the performance of the various PD methods.

## Results and discussion

### Measurement errors (*σ*_*obs*_)

**Fig. 4:**
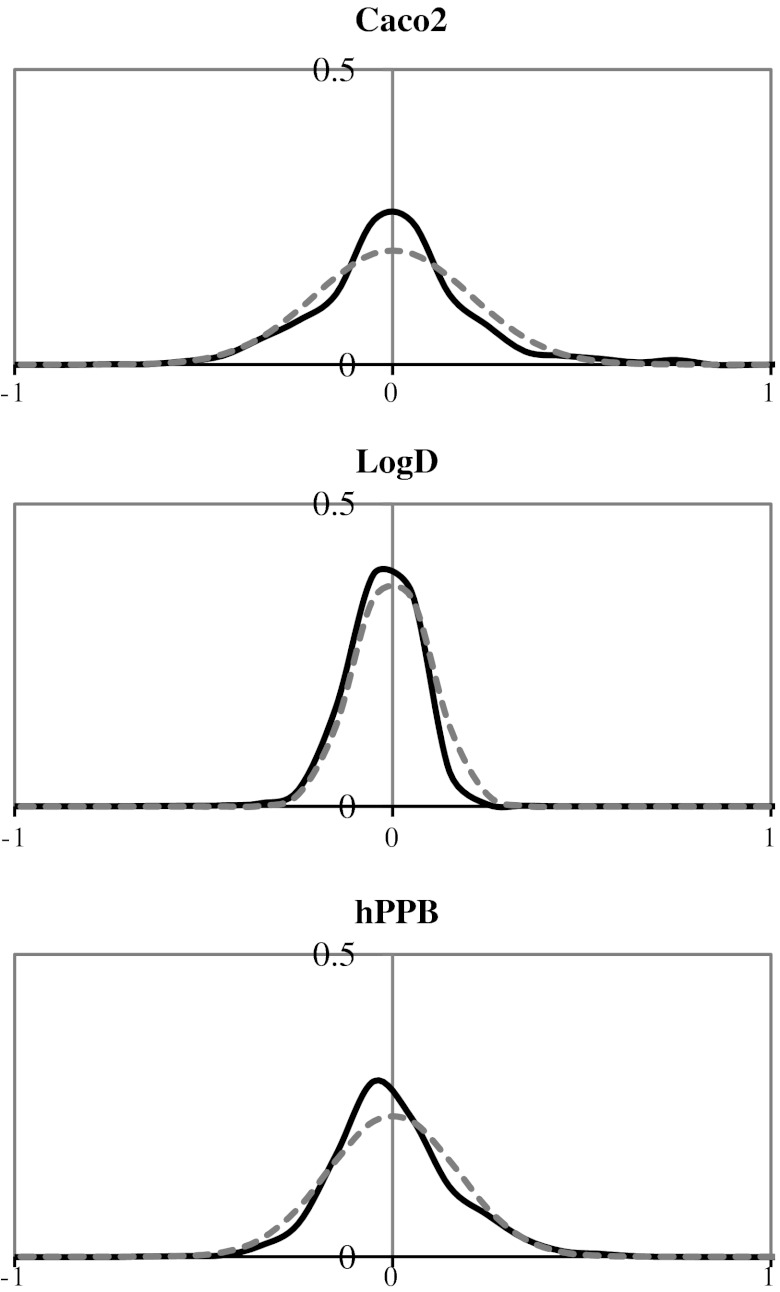
Normalized measurement error distributions obtained from the quality control compounds for each of the three endpoints

Figure 4 shows the normalized distributions of measurement values obtained from the QC compounds. The Caco2, LogD and hPPB assays had 3, 1 and 5 QC compounds, respectively. All distributions are shown in comparison to Gaussian distributions with the same standard deviation. Kolmogorov–Smirnov tests for normality revealed that all three of these distributions were significantly different from Gaussian distributions, but this result was unsurprising: there is no intrinsic reason why the measurement errors should be precisely normally distributed. Nonetheless, we felt that the distributions looked close enough to the Gaussians for it to represent a reasonable model for the errors. The standard deviations of the normalized measured values, and therefore the estimates of the experimental error, were 0.21, 0.10 and 0.17 for the Caco2, LogD and hPPB endpoints, respectively.

### Accuracy of the predictions

First, we compared the 4 modeling algorithms by the accuracy of their ‘data-point’ predictions of the combined experimental test set compounds using conventional QSAR validation techniques. Table 3 provides descriptive statistics of the experimental test set data for the 3 endpoints, and Table 4 summarizes the prediction accuracies of the 4 modeling algorithms. The SVM models produced the most accurate predictions and the RF models produced the second most accurate predictions for all three endpoints. The KNN algorithm was more accurate than the PLS algorithm for 2 of the 3 endpoints. The R_0_^2^ statistics for the predictions from the SVM models are 0.59, 0.73 and 0.63 for the Caco2, LogD and hPPB models respectively, which represents a reasonably high level of prediction accuracy for the studied endpoints.

**Table 3:** Descriptive statistics for the experimental test sets

	N	Mean	SD	Range	Expt error (*σ*_*obs*_)
Caco2	1,136	−5.47	0.74	−8.0 to −3.9	0.21
LogD	7,694	2.36	1.17	−4.0 to 5.8	0.10
hPPB	5,569	1.24	0.81	−1.4 to 4.3	0.17

**Table 4:** Comparison of the temporal test set (TS) and the double loop cross validation (CV) based estimate of the models’ forward prediction errors

Endpoint	Model	Obs. *R*_0_^2^	Obs. RMSE	TS $\widehat{RMSE}$	CV $\widehat{RMSE}$
Caco2	KNN	0.43	0.56	0.57*	0.48
	PLS	0.38	0.59	0.58*	0.66
	RF	0.53	0.51	0.47*	0.46
	SVM	0.59	0.47	0.49*	0.43
LogD	KNN	0.61	0.73	0.74*	0.62
	PLS	0.61	0.73	0.72	0.73*
	RF	0.70	0.64	0.65*	0.55
	SVM	0.73	0.61	0.63*	0.50
hPPB	KNN	0.56	0.53	0.50*	0.44
	PLS	0.52	0.56	0.59	0.53*
	RF	0.62	0.49	0.47*	0.41
	SVM	0.63	0.49	0.45*	0.37

*Obs. RMSE* and *Obs. R*_0_^2^ represent the prediction accuracy of the models against the experimental test sets. The $TS\,\widehat{RMSE}$ column provides estimate of the models’ forward generalization error based on the 2011-Q1 parameterization test sets. The $CV\widehat{RMSE}$ column provides estimates of the forward generalization error calculated with double loop cross validation on the training set. The uniform error estimation method that provided the closest estimate of the prediction error on the experimental test set is marked with an asterisk

Table 4 also provides the cross-validation $(CV\widehat{RMSE})$ and temporal test set $\left( {TS\,\widehat{RMSE}} \right)$ based estimates of the prediction error for comparison against the actual errors observed for the experimental test set (*Obs. RMSE*). In most cases, the TS-based estimations of the models’ expected prediction errors (generated using the 2011-Q1 dataset) are closer to the observed experimental test set error than the CV-based estimate. For the KNN, RF and SVM algorithms, the CV-based estimate always underestimated the prediction error of the models on the experimental test sets. For the SVM models, this underestimation is quite marked.

Any QSAR model validation should test the model with the kind of chemical structures that will be applied to the model in real life usage. When validating a model with cross validation there is therefore an implicit assumption that the range of compounds that comprise the model’s training set are representative of the kind of structures that will be applied to the model in its real-life use. Many pharmaceutical datasets, however, have an intrinsic temporal ordering. Typical compounds for which predictions are requested will be most similar to the more recent training set compounds and will often represent a move into a previously unexplored area of chemical space that stretches the model’s AD. This effect is supported by the results in Table 3. A key disadvantage of temporal test set approaches is that they use relatively small subsets of the dataset to validate models, and as a consequence, test set-based estimates of generalization error have a greater sampling error relative to the CV-based estimates. Furthermore, the range of chemistries represented by small temporal test sets may not cover the full range of chemistries that will be applied to the model. The temporal test sets used in the analyses presented in this paper are sufficiently large to alleviate these problems, and the test set-based method appears to better reflect the future prediction accuracies. These findings provide some evidence in favor of temporal test set-based model validation methods over internal validation procedures for quantitative pharmaceutical datasets. However, it should be emphasized that these datasets, which represent a sequential exploration of chemical space, are quite distinct from typical literature datasets that usually contain static and unordered data. Herein we will use the TS error estimation method as the benchmark for success for the variable error estimation methods.

### Parameterization of the error estimation methods

The D2M, LEC and BVC error estimation methods required up to three parameters to be set to convert the reliability score into a quantitative estimate of expected prediction error. These parameters were *m* and *c,* the gradient and intercept for the regression between the reliability indicator and the squared or absolute expected error, and *k,* the number of training set neighbors used to calculate the reliability scores. We fixed the parameter *c* to be the experimental error of the assays. The other parameters were optimized using the predictions obtained for the initial parameterization temporal test set for each of the algorithms and endpoints. Table 5 summarizes the optimal parameter values for *k*, which is the number of near neighbors used to calculate the reliability metric. The notation for the error estimation method in this table is {Method}-{DistanceMetric}; for example, D2M-EUC represents distance-to-model based error estimates with the Euclidean distance function, and LEC-MD indicates the Corrected Local Error method with the Mahalanobis distance function. The choice of distance metric (Euclidean or Mahalanobis distance) had very little effect on the optimal value for this parameter; the optimal values were between 1 and 5 for the D2M reliability methods, and between 50 and 200 for the local error-based methods.

**Table 5:** Range of optimal values of the parameter *k* for the various reliability methods

Error estimation method	KNN	PLS	RF	SVM
D2 M-EUC	2–3	12–50	2–5	1
D2 M-MD	2–3	8–50	1–3	1
LE-EUC	50–200	200	200	200
LE-MD	200	100–200	100–200	200
LEC-EUC	100–200	100–200	50–200	200
LEC-MD	100–200	12–200	50–200	100

### Performance of the PD methods

**Fig. 5:**
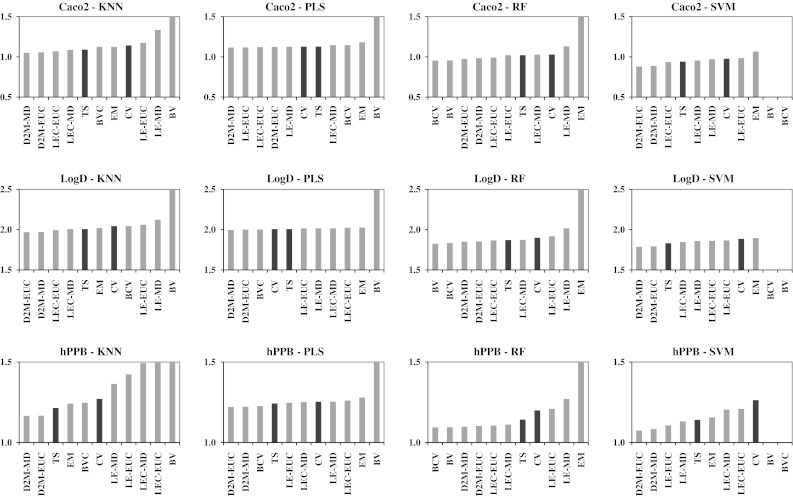
Charts showing the order of performance of the PD methods for each model/endpoint in terms of mean KL divergence against the combined experimental test sets. Variable error estimation methods are shown in *grey* and uniform error estimation methods are shown in *black*. The variable error estimation methods must result in a reduced mean KL divergence for them to represent an improvement on the uniform error estimation methods

Table 6 summarizes the performances of the PD methods across the three endpoints, as evaluated within the KL framework. We have italicized the uniform error methods and marked with an asterisk the best variable error estimation method for each modeling algorithm/endpoint. Any methods that performed better than the best uniform method are shown in bold. Figure 5 also provides a graphical representation of these results. The magnitudes of the mean KL divergence numbers are dependent on the magnitude of the estimated experimental measurement errors and the models’ prediction errors. This explains why the mean KL divergence numbers are highest for LogD, despite the fact that LogD models were most accurate as judged by the R_0_^2^ statistic. The D2M error estimation methods performed consistently well across all modeling algorithms and endpoints, and tended to result in mean KL divergences that are at least close to the best method. The Euclidean and Mahalanobis distance functions performed very similarly, with the Euclidean distance function typically resulting in slightly lower mean KL divergences. The BV and BVC methods performed very well when used in conjunction with the RF modeling algorithm, but less well when used in conjunction with the other modeling algorithms. The predictive distributions from the PLS:BV method resulted in particularly high KL divergences. This is because the individual models in the bagged ensembles are stable, high-bias, low-variance models, which caused the individual predictions to vary very little across the ensembles. As a consequence, the uncorrected BV method produced overly tight estimations of prediction errors for the PLS models. A similar effect was seen with the KNN:BV PD method, but to a lesser degree. In general, the other variable error estimation methods did not provide information gain relative to the uniform error estimation methods.

**Table 6:** Mean KL divergences for the various PD methods

	Caco2				LogD				hPPB			
	KNN	PLS	RF	SVM	KNN	PLS	RF	SVM	KNN	PLS	RF	SVM
*CV*	*1.14*	*1.13*	*1.03*	*0.98*	*2.04*	*2.01*	*1.90*	*1.89*	*1.27*	*1.25*	*1.20*	*1.26*
*TS*	*1.09*	*1.13*	*1.02*	*0.94*	*2.01*	*2.01*	*1.87*	*1.83*	*1.21*	*1.24*	*1.14*	*1.14*
D2 M-EUC	**1.06**	1.12*	**0.98**	0.88*	1.97*	**2.00**	**1.85**	1.79*	1.17*	1.22*	**1.10**	1.08*
D2 M-MD	1.05*	1.12*	**0.98**	**0.89**	1.97*	1.99*	**1.85**	1.79*	1.17*	1.22*	**1.10**	1.08*
LE-EUC	1.17	1.12*	1.02	0.98	2.06	2.02	1.92	1.87	1.42	1.25	1.21	**1.11**
LE-MD	1.33	1.12*	1.13	0.97	2.12	2.02	2.02	1.86	1.36	1.25	1.27	**1.13**
LEC-EUC	1.07	1.12*	**0.99**	0.94	**1.99**	2.02	1.87	1.86	1.53	1.26	**1.11**	1.21
LEC-MD	1.09	1.15*	1.03	0.95	2.01	2.02	1.87	1.84	1.49	1.25	**1.11**	1.20
BV	2.63	4.06	**0.96**	–	6.43	25.50	1.82*	–	3.27	5.36	**1.10**	–
BVC	1.13	1.15	0.95*	–	2.04	2.00	**1.83**	–	1.25	1.23	1.09*	–
EM	1.13	1.18	3.06	1.07	2.02	2.03	19.38	1.89	1.24	1.28	4.18	1.16

The uniform error estimation methods are italicized, and the best error estimation method for each model/endpoint is marked with an asterisk. Any variable error estimation methods that performed better than the best corresponding uniform method are shown in bold

We assessed the statistical significance of the results by fitting a linear mixed-effects model to the mean KL divergence numbers for the monthly experimental test sets. Endpoints and months were treated as random effects, and the algorithm and error estimation method as fixed effect. In essence we regard the endpoints and months as nuisance factors which we want to control for, whereas our main interests are on differences between algorithms and error estimation methods. Our aim for this analysis was to identify variable error methods that resulted in a statistically significant improvement to information relative to the corresponding uniform TS error estimation method, to a 95 % level of confidence. The linear mixed-effects model was fit using Gibbs sampling with the Bayesian software package JAGS [41]. Bayesian *P* values were calculated from the Gibbs sample using 10000 iterations. We used locally uniform priors for the fixed effects, and locally non-informative priors for the random effects. The mean KL divergence was assumed to follow a t-distribution, which was used instead of a normal distribution to account for the heavy tails of the distribution of mean KL divergence. The table of data that was used as an input for this analysis is provided in the supplementary information.

Table 7 provides the Bayesian *P* values for the variable PD methods that were determined to result in a statistically significant improvement to the information content of the predictive distributions. The D2M-EUC and D2M-MD-based PD methods resulted in a statistically significant improvement, relative to the uniform TS method, for all four modeling algorithms. Additionally, the RF:BV and RF:BVC, and RF:LEC-EUC methods resulted in a statistically significant improvement relative to the RF:TS method. For each modeling algorithm, we have compared 9 variable error estimation methods to a single uniform error estimation method; we therefore wondered whether the α-value for 95 % significance should be adjusted to account for multiple testing effects. Using a Bonferroni correction [42], significance at a 95 % level of confidence corresponds to a *P* value of $\frac{0.05}{9} = \, 0.00 5 6$. This seemed an excessively cautious adjustment as there is a high degree of correlation between the results across the different modeling algorithms and endpoints. However, even with this Bonferroni correction, most of the significant results remain significant. From this analysis we have concluded that the D2M error estimation method works consistently well across a range of different modeling algorithms, and results in a statistically significant improvement to the information content of the predictive distributions. Two PD methods stood out as particularly successful, which are the SVM:D2M-EUC method and the RF:BV method. We take a closer look at these methods in the remainder of the paper.

**Table 7:** A list of PD methods that resulted in a statistically significant improvement relative to their equivalent uniform TS method

Algorithm	Error method	Effect estimate	Bayesian *p* value
KNN	D2M-EUC	0.027	<0.001
KNN	D2M-MD	0.031	<0.001
PLS	D2M-EUC	0.007	0.045
PLS	D2M-MD	0.010	<0.001
RF	BVC	0.043	<0.001
RF	BV	0.045	<0.001
RF	D2M-EUC	0.025	<0.001
RF	D2M-MD	0.028	<0.001
RF	LEC-EUC	0.016	0.049
SVM	D2M-EUC	0.045	<0.001
SVM	D2M-MD	0.043	<0.001

Bayesian *P* values were calculated with a linear mixed-effects model

### Validity of the predictive distributions Gaussian assumption

**Fig. 6:**
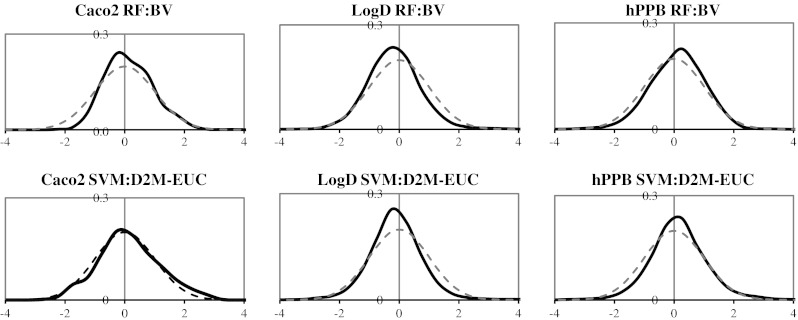
The validity of the predictive distributions shown graphically; the shape of the normalized prediction error distribution compared to Gaussian distributions

The current implementation of the KL framework assumes that the prediction errors are distributed as a Gaussian around the mean prediction value, and if the actual distributions of errors differ greatly from the Gaussian assumption, we may be able to improve on the KL divergence by using alternative functional forms for the error distribution. The histograms in Fig. 6 show the normalized distributions of predictions errors with the RF-BV, and SVM:D2M-EUC PD methods. We calculated the normalized prediction errors as $\left( {\mu_{obs} - \mu_{pred} } \right)/\sigma_{pred}$, which is the residual error divided by the estimated prediction error. Again, a Kolmogorov-Smirnov test for normality indicated that all distributions were significantly different from Gaussian distributions, but we felt that they were close enough to provide a useful model for the predictions errors.

### Behavior of KL divergence

**Fig. 7:**
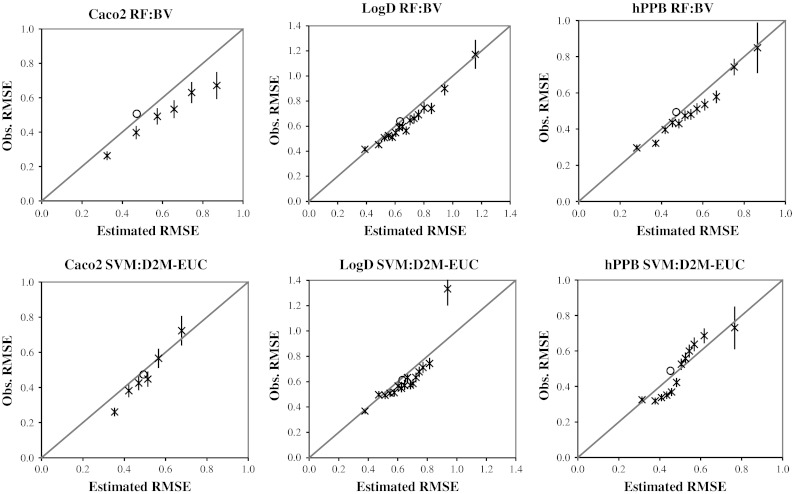
The accuracies of the prediction error estimates for the RF:BV and SVM:D2M-EUC PD methods. The predictions for the experimental test sets are grouped into groups with similar estimated prediction errors. The Caco2 data points represent bins of 200 compounds whereas the LogD and hPPB represent bins of 500 compounds. Error bars show the 95 % confidence intervals

Figure 7 provides a graphical representation of the accuracies of the prediction error estimates. We produced these charts by ranking the experimental test set predictions by their estimated errors (*σ*_*pred*_) and binning the predictions so that each bin contained test set compounds with similar estimated errors. We used a bin size of 200 for the Caco2 predictions and a bin size of 500 for the LogD and hPPB predictions. The *y* axis error bars represent the 95 % confidence interval for the RMSE and were calculated using Faber’s distribution-based approximation of the variance [43] (Eq. 8).

8 $$ \sigma \widehat{RMSE}/RMSE \approx \left( {1/2n} \right)^{\frac{1}{2}} $$

For each of the bins, the estimated and observed RMSEs correlate to the line of unity extremely well, and this shows that the RF:BV and SVM:D2M-EUC methods have provided error estimates that are a very good reflection of the actual prediction errors. The performance of the RF:BV method is particularly notable because the error estimate is obtained directly from the RF models with no further calculations required. The SVM:D2M-EUC method provided predictive distributions with the lowest KL divergence, but the computation time for both the SVM algorithm and the D2M-EUC error estimation method means that the SVM:D2M-EUC method is much more computationally expensive than the RF:BV method, both for model generation and for making predictions.

**Fig. 8:**
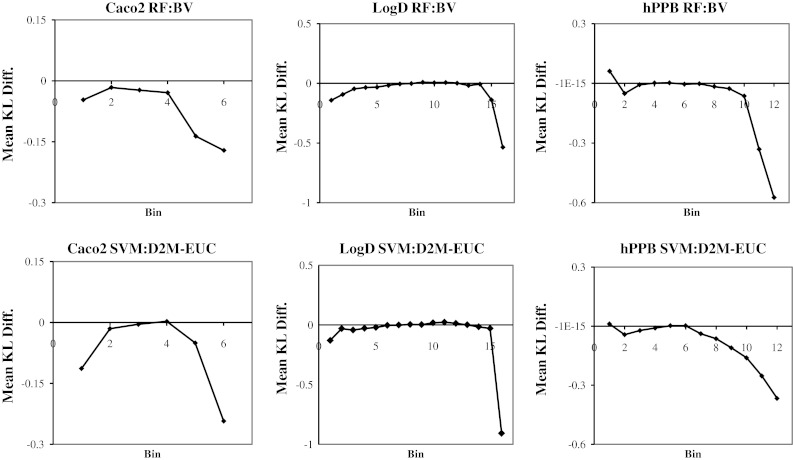
The difference between the best variable PD methods and the uniform TS PD method. The same binning scheme is used as in Fig. 7, i.e., the first bin represents the set of predictions with the lowest estimated prediction error and the last bin corresponds to the subset of predictions with the highest estimated prediction error. A negative Mean KL difference indicates that the variable method has increased information relative to the uniform TS method

Figure 8 provides charts that show the difference in the mean KL divergences relative to the equivalent uniform TS method for each of the bins shown in the charts in Fig. 7. The first data point in each of these charts represents the experimental test set predictions with the lowest estimated prediction errors, and the last data point represents the predictions with the highest estimated prediction errors. A negative KL difference indicates that the variable error estimation method has provided an information gain relative to the uniform TS method for the bin. In general the mean KL numbers behave exactly as we expected: the difference in the mean KL divergences is greatest for the bins at the two extremes. Reassuringly the shapes of the charts are consistent across endpoints, and the KL differences are negative or close to zero in almost all cases. The greatest reductions in the mean KL divergences from the variable error estimation methods are seen in the bins corresponding to the predictions with the highest estimated error, which suggests that the majority of the information gains are achieved by recognizing the compounds that are likely to result in poor predictions.

In Fig. 8, the SVM:D2M-EUC and RF:BV charts for hPPB both feature a positive spike in the mean KL difference in the first bin. These spikes are caused by a small number of predictions that were estimated to have a very low error, but that were badly mispredicted. These predictive distributions resulted in very high KL divergences and this skewed the mean KL divergence for the bin. The compounds behind these predictions all featured multiple stereo-centers, but the descriptor set used to encode the structures for the QSAR modeling is achiral. Leach et al. [44] have recently shown that protein binding is affected by stereochemistry. The mispredictions were caused by diastereoisomers with identical descriptor representations but different 3-dimensional shapes, and therefore different levels of protein binding. Essentially, the RF and SVM models would match these compounds to previously seen isomers, and, with a high degree of confidence, incorrectly predict the protein binding to be the same as the previous compound. This observation highlights limitations of models built with achiral descriptors and reveals a specific type of model applicability domain error.

### Probability of hitting a target profile

Given valid predictive distributions, we can convert predictive distributions into estimates of the probability that an untested compound has properties that match a desired target profile (TP). Probabilistic approaches similar to this have previously been explored by Segall et al. [45–47]. The approach provides a flexible alternative to classification models because the threshold values for the properties can be defined by users at the point of prediction, rather than when the model is generated. Furthermore, we believe that expressing predictions as probabilities provides an intuitive way of representing prediction errors to the model users [48]. Expressing QSAR predictions as probabilities also allows users to make intelligent decisions about the numbers of compounds that must be synthesized to stand a reasonable chance of producing a compound that meets the project’s requirements. Information like this can potentially be used to prioritize synthetic chemistry resources towards projects that do not have access to reliable QSAR predictions. In this section, first we will consider target profiles that comprise a single drug optimization parameter, and then will give a couple of examples of probability estimates for 2-parameter target profiles. We do not have enough compounds spanning all three of the datasets to extend the analysis beyond 2 optimization parameters. We also wish to emphasize that all these target profile ranges are arbitrary and are intended only to demonstrate the approach.

**Fig. 9:**
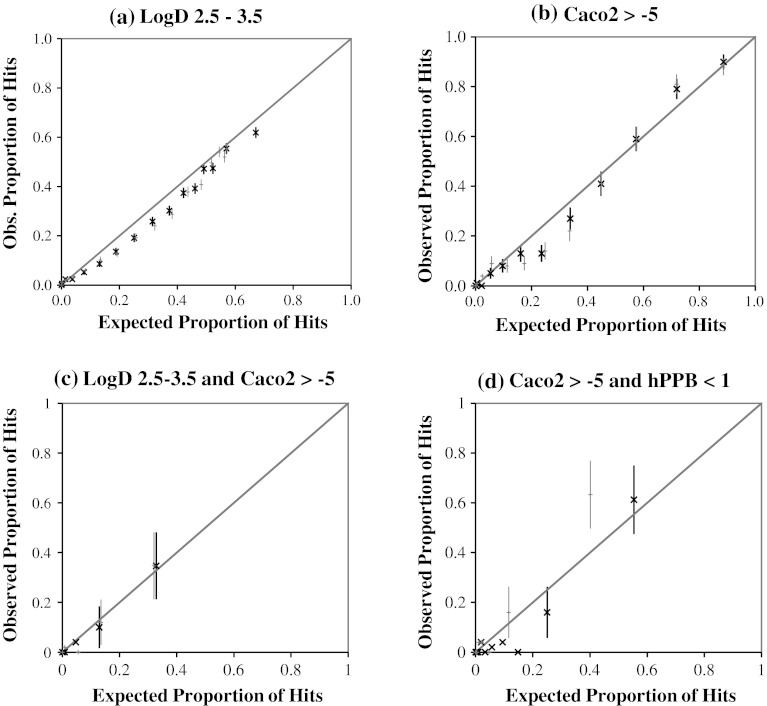
Calibration plots for the estimated probabilities that a test compound hits a target profile. Charts **a** and **b** are single-objective target profiles and charts **c** and **d** are double-objective target profiles. The main *black* data points represent the SVM:D2 M-EUC model’s predictions. The *grey* data points show equivalent results obtained from the SVM:TS method for comparison. *Error bars* on the y-axis show the 95 % confidence interval of the mean and are calculated using a central limit theorem approximation (*error bars* are shown only if this approximation is reasonable, i.e., the number of hits in the sample is greater than 5)

The accuracies of the probability estimates for each of the target profile datasets are shown graphically with calibration plots in Fig. 9 [49]. We produced the calibration plots, which are similar to the plots shown in Fig. 7, by ranking and binning the compounds according to their estimated probabilities of hitting the target profiles so that each bin contains compounds with similar probability estimates. We used the SVM:D2M-EUC PD method for all these plots as it was the method that produced predictive distributions with the highest information content. Equivalent results obtained using the SVM:TS method are shown in grey for comparison. A high correlation of the data points with the line of unity indicates that the probability estimates are an excellent reflection of the actual observed probabilities. The *y* error bars were calculated with central limit theorem and show 95 % confidence intervals on the data points.

The first target profile is a LogD within the range 2.5–3.5. We ranked the 7698 predictions from the LogD experimental test sets and calculated the proportion of each predictive distribution that lies within the target profile range with a Gaussian cumulative distribution function (CDF) using the predicted *μ*_*pred*_ and *σ*_*pred*_ values. The calibration plot in Fig. 9a shows a good agreement between the estimated probabilities and the actual observed likelihood of hitting the target profile. There is a slight bias to the probability estimates, with the probabilities of hitting the target profile consistently overestimated; however, we feel that this bias is small enough that it should not significantly impact the model users. The most obvious difference between the results from the SVM:D2M-EUC method and the more simple SVM:TS method lie in the highest probability bin, indicating that the SVM:D2M-EUC method has been able to identify some of the hits with a higher degree of confidence than the SVM:TS method. Nonetheless, both methods provide reasonable probability estimates. The second target profile is for a Caco2 cell permeability of >−5 (log cm/s). Again, the chart in Fig. 9b shows that the probability estimates are an accurate reflection of the likelihood of a test compound hitting the target profile. In this case, there is no clear difference between the SVM:D2M-EUC and SVM:TS methods.

The third and fourth target profiles considered here feature two optimization parameters. Target profile 3 is defined as LogD in the range 1.5–2.5 and Caco2 permeability of greater than >−5 (log cm/s). There were only 225 compounds common to both the LogD and Caco2 permeability experimental test sets, and only 24 (11 %) of these compounds hit the 2-parameter target profile. We calculated the probability of a compound having the desired target profile by multiplying the probability estimates for individual components, each of which were calculated with the Gaussian CDF function as described above. We produced the calibration plot shown in Fig. 9c with a bin size of 50, and it shows a high correlation between the estimated and observed hit rates. The fourth 2-parameter target profile is Caco2 >−5 (log cm/s) and hPPB <1 (log bound/free). In total, 546 compounds were common to both experimental test sets and 43 (8 %) of these compounds hit the target profile. The calibration plot shown in Fig. 9d was produced with a bin size of 50 compounds. Again, the estimated probabilities match the observed proportions of hits very well, although we do wish to emphasize that both these 2-parameter target profiles were based on limited data with very few TP hits and the performance on these two TPs may not be generalizable to all other 2-parameter target profiles. In this final case, the SVM:D2M-EUC method provided more accurate probability estimates than the SVM:TS method.

Our focus in this paper is on providing the best possible information to model users for individual predictions, rather than improving the rankings of large sets of compounds. However, at this point we should note that the differences in the performance of the SVM:D2M-EUC, SVM:TS, RF:BV and RF:TS methods, in terms of ranking of the TP hits, are quite small. The differences between the rankings for the uniform and variable methods are most obvious for the highest ranked TP hits; for example, in the LogD 1.5-2.5 profile above, the SVM:D2M-EUC method ranked 384 TP hits in the first 500 compounds, compared to 348 hits for the SVM:TS method; and the RF:BV method ranked 381 TP hits in the first 500 compounds, compared to 309 with the RF:TS method. The data used for these analyses are provided as supplementary information.

## Summary and conclusions

We have outlined an information theoretic framework for assessing QSAR predictions based on KL divergence, in which both predicted and experimentally measured properties are treated as Gaussian-shaped probability distributions. By treating QSAR predictions as probability distributions, estimations of error become intrinsic to the predictions themselves and error estimation becomes an integral part of the model generation and selection process. The ‘models’ in this work consisted of two components: (1) a modeling algorithm that assigns the means the prediction values, and (2) an error estimation method that assigns a quantitative value to the error of the prediction (the width of the Gaussian predictive distribution).

We chose to use KL divergence to quantify the distance between probability distributions because of its grounding in maximum likelihood theory and because it is probably the most widely used metric for comparing probability distributions. Other metrics, such as a simple overlap score, may provide a more intuitive result, although we do feel that the KL numbers will become more intuitive with increased usage and familiarity. Further work could be aimed at assessing alternative metrics for quantifying the distance of probability distributions.

Using the KL framework, we assessed a range of different predictive distribution models in a time-series study that spanned 1 year’s worth of AstraZeneca’s data for 3 global DMPK assays. Two predictive distribution methods stood out as particularly successful: (1) Support Vector Machine modeling algorithm with distance-to-model based error estimation, and (2) the Random Forests modeling algorithm with bagged variance-based error estimation. A statistical analysis of our data showed that these methods provided a significant improvement in information relative to ‘uniform’ error estimation methods, in which all test compounds are assigned the same error estimate. The Random Forest bagged variance method is of particular note because excellent error estimations can be obtained directly from the Random Forest models with no extra calculations required.

Throughout this work we have assumed that predictions and measurement errors have a Gaussian-shaped distribution. This assumption is commonplace in statistics and, after inspecting the actual error distributions for predictions and experimental measurements, we feel that it is a practical and useful model for the errors. Nonetheless, the Gaussian assumption is not a requirement for the KL framework and alternative error distribution models may be more suitable. Any alternative error distributions can be assessed alongside Gaussian error distributions within the framework.

With methods that produce valid predictive distributions, we can estimate the probability that a virtual, untested compound has properties that match a desired target pharmacological profile. We have shown that our best methods can produce accurate probability estimates for both single and multi-objective target profiles. We feel that presenting predictions in this manner represents prediction errors in a way that is intuitive, and may allow strategic allocation of synthetic chemistry resources to projects that do not have access to accurate predictive models.

In future work we will investigate methods for assigning non-parametric predictive distributions that do not require an assumed functional form. We will also apply the predictive distribution methods described in this paper to local datasets to determine whether they are able to recognize completely out-of-domain prediction queries.

## Electronic supplementary material

Below is the link to the electronic supplementary material.

Supplementary material 1 (XLSX 75 kb)

Supplementary material 2 (XLSX 14 kb)

Supplementary material 3 (XLSX 2637 kb)
